# Comprehensive molecular characterization of hypertension-related genes in cancer

**DOI:** 10.1186/s40959-022-00136-z

**Published:** 2022-05-05

**Authors:** Yanan Jiang, Chunpeng Shi, Songyu Tian, Fengnan Zhi, Xiuyun Shen, Desi Shang, Jinwei Tian

**Affiliations:** 1 Department of Pharmacology (State-Province Key Laboratories of Biomedicine- Pharmaceutics of China, Key Laboratory of Cardiovascular Research, Ministry of Education), College of Pharmacy, Harbin Medical University, Harbin, 150081 China; 2 Translational Medicine Research and Cooperation Center of Northern China, Heilongjiang Academy of Medical Sciences, Harbin, 150081 China; 3 Department of Gynecology, Harbin Medical University Cancer Hospital, Harbin, 150081 China; 4 The First Affiliated Hospital, Hengyang Medical School, University of South China, Hengyang, 421001 Hunan China; 5 Department of Cardiology, The Second Affiliated Hospital of Harbin Medical University, Harbin, 150086 China; 6 The Key Laboratory of Myocardial Ischemia, Ministry of Education, Harbin Medical University, Harbin, 150086 China

**Keywords:** Hypertension-related genes, Pan-cancer, Cardio-oncology

## Abstract

**Background:**

During cancer treatment, patients have a significantly higher risk of developing cardiovascular complications such as hypertension. In this study, we investigated the internal relationships between hypertension and different types of cancer.

**Methods:**

First, we comprehensively characterized the involvement of 10 hypertension-related genes across 33 types of cancer. The somatic copy number alteration (CNA) and single nucleotide variant (SNV) of each gene were identified for each type of cancer. Then, the expression patterns of hypertension-related genes were analyzed across 14 types of cancer. The hypertension-related genes were aberrantly expressed in different types of cancer, and some were associated with the overall survival of patients or the cancer stage. Subsequently, the interactions between hypertension-related genes and clinically actionable genes (CAGs) were identified by analyzing the co-expressions and protein–protein interactions.

**Results:**

We found that certain hypertension-related genes were correlated with CAGs. Next, the pathways associated with hypertension-related genes were identified. The positively correlated pathways included epithelial to mesenchymal transition, hormone androgen receptor, and receptor tyrosine kinase, and the negatively correlated pathways included apoptosis, cell cycle, and DNA damage response. Finally, the correlations between hypertension-related genes and drug sensitivity were evaluated for different drugs and different types of cancer. The hypertension-related genes were all positively or negatively correlated with the resistance of cancer to the majority of anti-cancer drugs. These results highlight the importance of hypertension-related genes in cancer.

**Conclusions:**

This study provides an approach to characterize the relationship between hypertension-related genes and cancers in the post-genomic era.

**Supplementary Information:**

The online version contains supplementary material available at 10.1186/s40959-022-00136-z.

## Introduction

Cancer is a major health problem and the second leading cause of death globally [1]. During cancer treatment, patients have a significantly higher risk of developing cardiovascular complications such as hypertension [2].

The antiangiogenic drugs sunitinib, sorafenib, and pazopanib pose greater risks of inducing hypertension compared with controls (everolimus, placebo, and interferon-α) [3, 4]. The application of cabozantinib is associated with the risk of developing hypertension [5]. Similarly, a recombinant humanized monoclonal IgG1 antibody called bevacizumab could also increase the risk of hypertension [6]. Santoni et al. reported that patients with solid tumors receiving targeted therapy (VEGFR/EGFR tyrosine kinase inhibitors) have the highest risk of hypertension events [7]. On the other hand, hypertension is related to an increased risk of cancer. Largent et al. observed that the long-term use of antihypertensive medication increased the risk of invasive breast cancer [8]. Choi et al. found that hypertension increased the mortality risk of kidney cancer in a cohort of Korean men [9]. Therefore, close monitoring and appropriate management for hypertension are strongly recommended during cancer treatment.

These studies suggest there may be an intrinsic interaction between cancer and cardiovascular diseases such as hypertension. Some hypertension-inducing genes have also proved to play critical roles in the pathogenesis of cancers. For example, low methylation of the α-adducin (*ADD1*) gene promoter increases the risk of essential hypertension [10], and the variation of *ADD1* is associated with the risk of colorectal cancer [11].

However, previous studies mainly focused on single genes in a few types of cancer. The molecular portrait of hypertension-related genes in cancer has not been comprehensively characterized, so the correlation between cancer and hypertension remains a major blind spot. Therefore, in the present study, we comprehensively characterized the role of hypertension-related genes in various cancers using multidimensional ‘‘omic’’ data from the cancer genome atlas (TCGA) database.

## Methods

### Somatic copy number alteration analysis

TCGA thresholded copy number alteration (CNA) scores for 9,125 patient samples were obtained from Genome Data Commons [12]. Significantly amplified and deleted genes (q < 0.25) were identified based on the copy number segmentation file in each cancer type using GISTIC2.0. Mutual exclusivity analysis was performed using the CoMET module [13] in R for hypertension-related genes in different types of cancer with *p* < 0.05.

### Somatic single nucleotide variant analysis

Somatic single nucleotide variant (SNV) data were obtained from Genome Data Commons. The filtering steps were selected according to a previous publication [14]. Subsequently, 19 genes from their corresponding bins were randomly selected to generate a random gene set with a similar length distribution as the hypertension-related genes. The frequencies of SNV changes of this randomly sampled gene set were calculated. After repeating the sampling 1,000 times, the ranks of the hypertension-related genes’ DNA aberration frequencies given the random sampling background were calculated.

### Gene expression analysis

Normalized gene expression data were obtained from Genome Data Commons [12]. Paired student’s *t*-tests were performed to calculate differentially expressed genes in cancer versus normal samples. A false discovery rate (FDR) with q < 0.05 was considered statistically significant. All negative values were considered as missing values (NA). The log-transformed value was used in the analysis.

### Interactions and correlations between hypertension-related genes and CAGs

The method used to identify clinically actionable genes (CAGs) is described in our previous work [15]. CAGs contain 135 target therapeutic genes (http://archive.broadinstitute.org/cancer/cga/target) [16] and 19 immunotherapeutic genes [17]. Pearson’s correlation coefficients (PCCs) of |R|> 0.3 and *p* < 0.05 were considered to indicate significant correlations between hypertension-related genes and CAGs. Protein–protein interaction (PPI) data were obtained from Human Protein Reference database (HPRD) [18] and database of protein, genetic and chemical interactions (BioGRID) [19].

### Patient survival analysis

Clinical data were obtained from Genome Data Commons [12]. The correlation between the hypertension-related genes and overall survival (OS) of patients was calculated [14]. Next, cancer purity data were collected from Genome Data Commons [12], and the above analysis was repeated while taking tumor purity as a Cox model covariate [14].

### Biological pathway analysis

The enriched biological pathways of hypertension-related genes were identified using the gene set enrichment analysis (GSEA) pre-ranked tool [20]. Briefly, each gene was ranked based on its expression correlation coefficient with the hypertension-related genes within each cancer type. The pre-ranked gene lists were then run against using GSEA Java (version 2.2.3). The normalized protein expression data (Z-score) from the TCGA RPPA platform were integrated into 11 core cellular pathways [21]. The Spearman’s rank correlations of the hypertension-related genes with these pathway scores were calculated. Additionally, q < 0.05 was considered statistically significant.

## Results

### Somatic alteration landscape of hypertension-related genes across 33 types of cancer

**Fig. 1:**
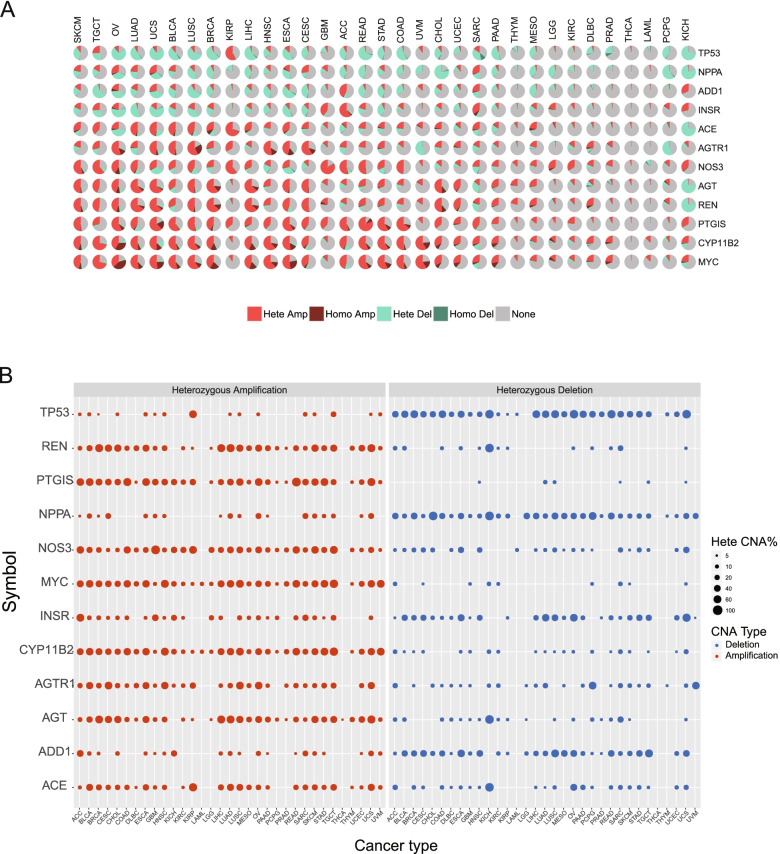
Somatic copy number alteration (CNA) of hypertension-related genes in different types of cancer. **A** The proportion of CNA of hypertension-related genes in different types of cancer. Light red and deep red represent heterozygous amplification (Hete Amp) and homozygous amplification (Homo Amp), respectively. Light green and deep green represents heterozygous deletion (Hete Del) and homozygous deletion (Homo Del), respectively. **B** Significant heterozygous amplification and deletion peaks of hypertension-related genes in each cancer type. Dot size represents the heterozygous percentage of CNA (Hete CAN %). Red and blue dots represent the amplification peak and deletion peak, respectively

Ten hypertension-related genes were curated from the literature (Table 1) [22]. The somatic CNA, SNV, and the corresponding mutation frequencies of these genes were calculated in the pan-cancer cohort. *TP53* served as a positive control in the following analyses. The CNA and driver mutation patterns of hypertension-related genes are shown in Fig. 1A. The heterozygous and homozygous expressions of hypertension-related genes were calculated across 33 cancer types. *CYP11B2*, *PTGIS*, *REN*, and *AGT* exhibited the highest amplification frequencies. *NPPA* had the most deep deletions. Regarding the mutational profile, *PTGIS* exhibited the most mutation frequencies in uterine carcinosarcoma. Thyroid carcinoma and acute myeloid leukemia had few somatic alterations in hypertension-related genes compared with other cancers.

**Table 1:** Hypertension-related genes

Gene ID	Gene symbol	Full name
5972	REN	Renin
1636	ACE	Angiotensin I converting enzyme
185	AGTR1	Angiotensin II receptor type 1
183	AGT	Angiotensinogen
1585	CYP11B2	Cytochrome P450, family 11, subfamily B, member 2
4846	NOS3	Nitric oxide synthase 3
5740	PTGIS	Prostaglandin I2 synthase
118	ADD1	Adducin 1
3463	INSR	Insulin receptor
4878	NPPA	Natriuretic peptide A

Significant amplification and deletion peaks of heterozygous hypertension-related genes were identified using GISTIC2.0 [23]. The heterozygous amplification and deletion states are shown in Fig. 1B, and the homozygous amplification and deletion states are shown in Fig. S1.

Additionally, the CNAs of hypertension-related genes were correlated with cancer. Among all 10 genes, *ADD1* was correlated with the most types of cancer, and it was especially strongly correlated with breast invasive carcinoma, head and neck squamous cell carcinoma, and lung squamous cell carcinoma. On the contrary, *AGTR1* was correlated with the fewest types of cancer (Fig. S2). These results showed that a highly heterogeneous/homozygous somatic alteration of hypertension-related genes was present across 33 cancer types.

**Fig. 2:**
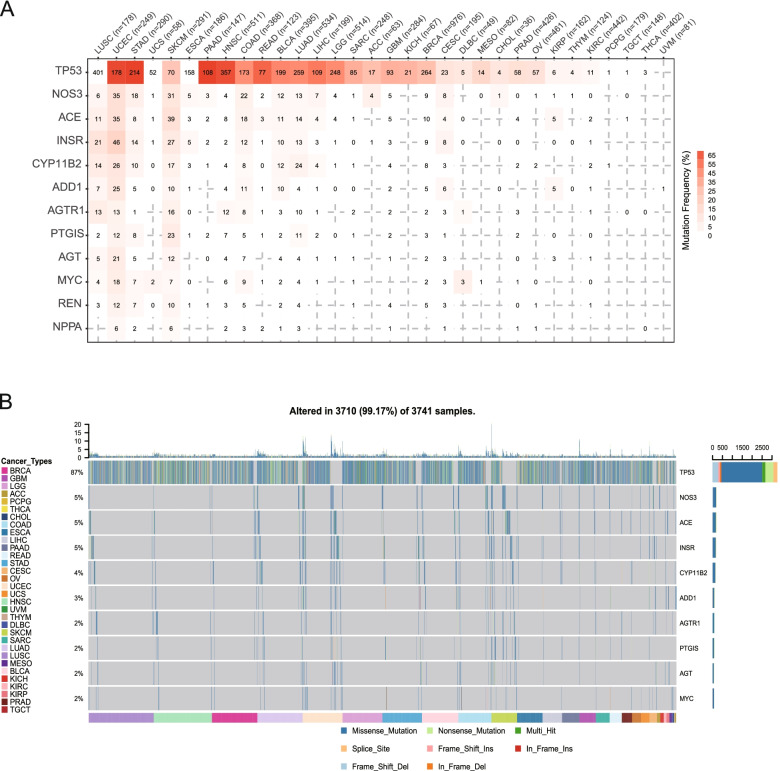
Single nucleotide variant (SNV) and mutation patterns of hypertension-related genes in cancer. **A**. Mutation frequency distribution of hypertension-related genes in each cancer type. The number in each box represents the number of mutation samples identified in the corresponding cancer type. Red intensity represents the percentage of mutation. **B** Waterfall plots of somatic alteration of hypertension-related genes in each cancer type. Genes are ranked from high to low SNV frequency. Ins and Del are short for insertion and deletion, respectively

Significantly mutated genes were identified using MutSigCV [24]. A random sampling approach was used to evaluate the alterations of hypertension-related genes in various types of cancer. Among the hyper-altered cancer types, uterine corpus endometrial carcinoma, skin cutaneous melanoma, and lung squamous cell carcinoma were the most prominent cancer types, which was probably due to the high mutation frequencies of *INSR*, *ACE*, and *NOS3* (Fig. 2A).

The mutation frequency was subsequently calculated in the pan-cancer cohort. The overall DNA aberration percentage ranged from 2% to 5%. *NOS3* showed the highest amplification frequency, followed by *ACE*, *INSR*, and *CYP11B2*. Missense mutation also frequently occurred in *NOS3* (Fig. 2B). SNV was also correlated with the overall survival of cancer patients. The mutation of *NOS3* was positively correlated with the overall survival of adrenocortical carcinoma patients. In contrast, the mutation of *ACE* was negatively correlated with the overall survival of skin cutaneous melanoma patients (Fig. S3). The SNV characteristics of hypertension-related genes in different types of cancer are summarized in Fig. S4.

### The expression pattern of the hypertension-related genes in cancer

**Fig. 3:**
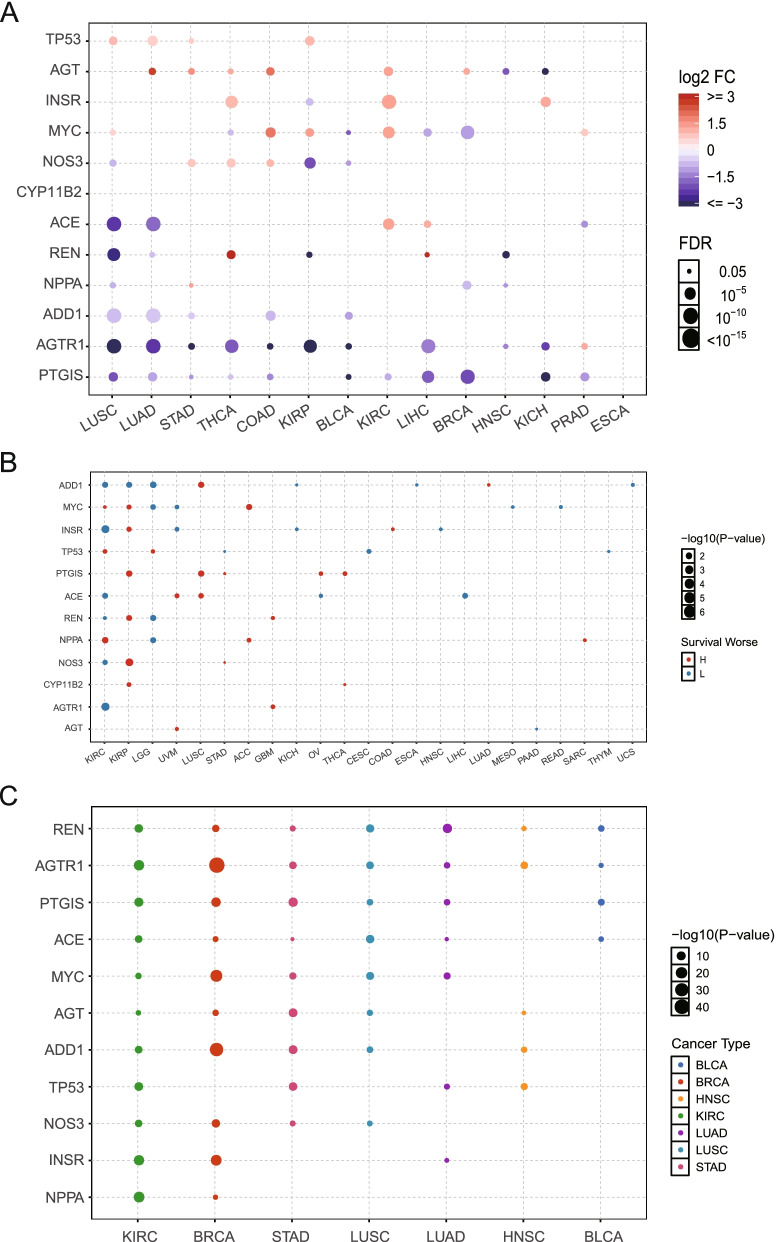
Expression pattern of hypertension-related genes in each cancer type. **A** Significant fold change peaks of hypertension-related genes in each cancer type. Dot size represents the magnitude of the false discovery rate. Red and blue dots represent upregulation and downregulation, respectively. **B** Correlations of hypertension-related genes with overall survival of patients for different cancer types. Dot size represents statistical significance. Blue and red represent negative and positive correlation with poor survival. **C** The association of hypertension-related genes with cancer stage. Dot size represents statistical significance. Dot colour represents different cancer types

The expression patterns of hypertension-related genes were analyzed for 14 types of cancer. Among them, lung squamous cell carcinoma had the largest number of differentially expressed hypertension-related genes, whereas esophageal carcinoma had none. Thus, esophageal carcinoma appears to have a very distinct carcinogenic mechanism from other kinds of cancer (Fig. 3A). A significant positive or negative correlation between hypertension-related genes and the overall survival of patients was observed in 23 cancer types (Fig. 3B). The association of hypertension-related genes with cancer stage is shown in Fig. 3C. *REN* was associated with the clinical stage of all cancers, including renal clear cell carcinoma, breast invasive carcinoma, stomach adenocarcinoma, lung squamous cell carcinoma, lung adenocarcinoma, head and neck squamous cell carcinoma, and bladder urothelial carcinoma. The stage of breast invasive carcinoma was correlated with *AGTR1* and *ADD1* (Fig. 3C).

### Interaction between CAGs and the hypertension-related genes

**Fig. 4:**
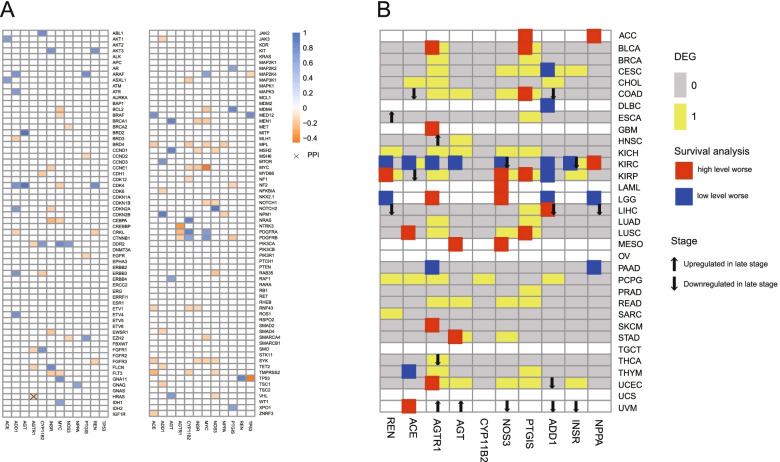
Clinical relevance of the hypertension-related genes. **A** Correlation between hypertension-related genes and clinically actionable genes (CAGs). Pearson’s correlation coefficient |R|> 0.3, *P* < 0.05. Red and blue boxes represent positive and negative correlation, respectively. Color scale represents the magnitude of Pearson’s correlation coefficient. The white boxes indicate the cancer type number is less than five. The “ × ” represents the protein–protein interactions (PPIs) of hypertension-related genes and CAGs based on the data from HPRD. **B** Clinical relevance of the hypertension-related genes across 33 cancer types. Yellow boxes indicate differently expressed hypertension-related genes in cancer. The up and down arrows represent the upregulation and downregulation of genes in later stages (III/IV) compared with early stages (I/II) (fold change > 1.5). Yellow (1) represent differentially expressed genes (DEGs), and Grey (0) represent non-DEGs. The red and blue boxes indicate high and low expression, respectively, in tumors associated with worse overall survival (log rank test FDR < 0.05)

To further reveal the clinical implications of the hypertension-related genes in cancer, the expression profiles of these genes were correlated with clinically actionable genes (CAGs). Gene pairs were screened based on PCC |R|> 0.3 and FDR < 0.05. The hypertension-related genes were correlated with CAGs. Among them, some protein–protein interactions were observed (Fig. 4A). The results showed that hypertension-related genes and CAGs had interactions. The data analysis was based on the protein–protein interaction data from HPRD [25] and the BioGRID database [26]. Therefore, the interaction between hypertension-related genes and CAGs may affect drug response.

### Clinical relevance of the hypertension-related genes

The association of interaction genes with the overall survival of patients and cancer stage was investigated. These genes were all associated with the overall survival and/or cancer stage in at least one cancer type. For example, INSR was downregulated in late stage of renal clear cell carcinoma, which was negatively correlated with worse prognosis of patients (Fig. 4B).

### Biological pathways associated with the hypertension-related genes

**Fig. 5:**
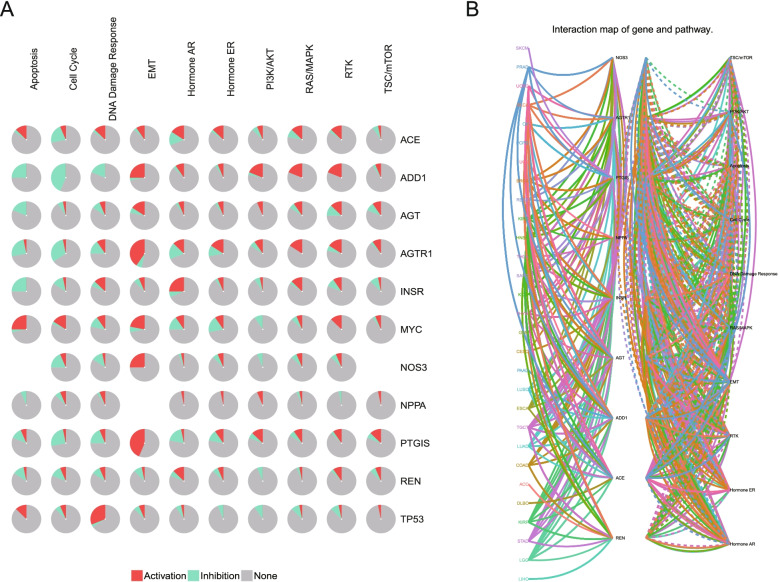
Biological pathways associated with hypertension-related genes. **A** The signaling pathways correlated with hypertension-related genes. Red and green represent activation and inhibition, respectively. **B** The correlation of hypertension-related genes and signaling pathways in each cancer type. The colour of lines represents different genes. Solid and dotted lines represent activation and inhibition, respectively. EMT: epithelial to mesenchymal transition; AR: androgen receptor; ER: estrogen receptor; RTK: receptor tyrosine kinase; RAS: renin-angiotensin system; TSC: tuberous sclerosis complex; mTOR: mammalian target of rapamycin

Next, we focused on gene-associated biological pathways. GSEA was performed based on the rank of gene expression levels. The positively correlated pathways included epithelial to mesenchymal transition (EMT), hormone androgen receptor (AR), and receptor tyrosine kinase (RTK), and the negatively correlated pathways included apoptosis, cell cycle, and DNA damage response (Fig. 5A). The signaling pathways correlated with hypertension-related genes for each cancer type are shown in Fig. 5B.

### Correlation between the hypertension-related genes and drug sensitivity of cancer

**Fig. 6:**
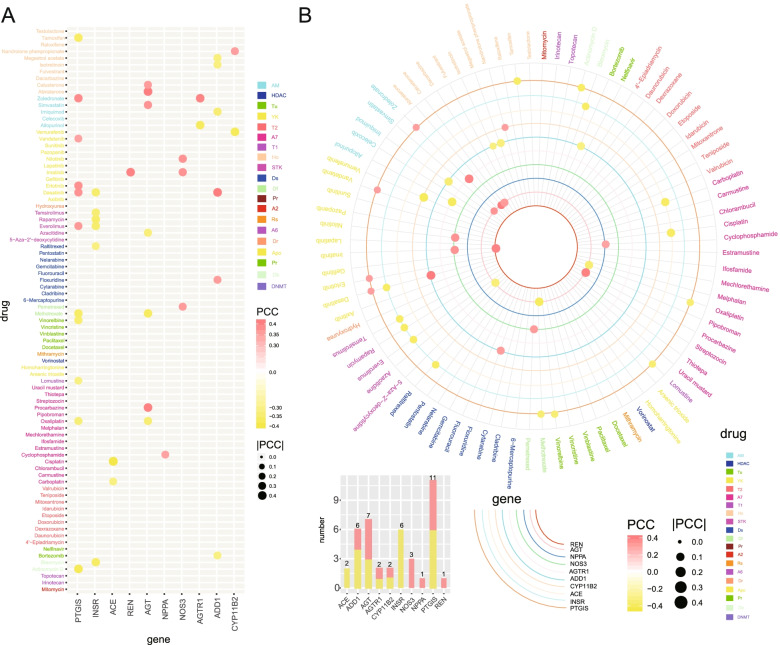
Correlation between the hypertension-related genes and drug sensitivity of cancer. **A** Correlation between hypertension-related genes and drug sensitivity. Red and yellow represent positive and negative correlation, respectively. The size of each dot represents the magnitude of Pearson’s correlation coefficient (PCC). The colour bars located along the *y*-axis indicate drugs and their category. **B** A circos chart showing the correlation between hypertension-related genes and drug sensitivity. Each circle represents a cardiovascular disease-related gene. Red and yellow represent positive and negative correlation, respectively. The size of each dot represents the magnitude of the Pearson’s correlation coefficient. The colour bars located on the bottom right of the figure indicate drugs and their category. Screen gene pairs with PCC |R|> 0.3 and false discovery rate (FDR) < 0.05

A total of 76 FDA-approved anti-cancer drugs were collected. The correlation between drug sensitivity and expression was analyzed for each of the hypertension-related genes. For example, NOS3 expression was positively correlated with the drug sensitivity of nilotinib, imatinib, and pemetrexed. While, ACE expression was negatively correlated with the drug sensitivity of cisplatin and carboplatin (Fig. 6).

## Discussion

Cardiovascular diseases are frequent complications of cancer patients. A new term called cardio-oncology has been introduced to describe the management of cancer patients with cardiovascular diseases. New approaches to prevent or manage cardiovascular side effects during cancer treatment are needed.

In this study, a comprehensive molecular characterization of the hypertension related genes across 33 types of cancer was performed, which involved 9,125 samples from the TCGA database. The somatic alteration landscape of the hypertension-related genes across 33 types of cancer was calculated.

The three genes with the highest amplification frequencies in cancer were *CYP11B2* (which encodes aldosterone synthase), *PTGIS* (which encodes prostacyclin synthase), and *Ren* (which encodes renin) (Fig. 1). The expression and therapeutic potential of *PTGIS* has been previously identified. In one study, reduced *PTGIS* expression was observed in human non-small cell lung cancer compared with that in controls [27]. In another study, overexpression of *PTGIS* inhibited lung cancer progression [28]. Additionally, *PTGIS* mRNA and protein expression level was found to be lower in head and neck carcinoma tissues, and low *PTGIS* mRNA expression was correlated with poor survival of head and neck carcinoma patients [29].

Some hypertension-related genes exhibited high deletion frequency, including *NPPA*, *ADD1*, and *INSR* (Fig. 1). The CNA of *ADD1* is correlated with nearly all cancer types, although it is most strongly correlated with breast invasive carcinoma (Fig. S2). *ADD1* expression was downregulated in lung adenocarcinoma tissues compared with that in normal lung tissues. The downregulation of *ADD1* promoted the migration of non-small cell lung cancer cells, and the overexpression of *ADD1* exerted the opposite effect [30]. However, the relevance of these genes, including *ADD1*, in breast invasive carcinoma has not been reported. Low-frequency CNA and SNV of hypertension-related genes was observed in thyroid carcinoma, suggesting that unknown epigenetic mechanisms were involved in controlling these genes. *NOS3*, *ACE*, and *INSR* have high mutation frequencies in most cancer types. None of the hypertension-related genes had single nucleotide variants in uveal melanoma, thyroid carcinoma, testicular germ cell tumors, or pheochromocytoma and paraganglioma.

We developed a signature of hypertension-related genes in predicting patient prognosis. The mutations of *NOS3* and *ACE* have prognostic potential for the overall survival of adrenocortical carcinoma and skin cutaneous melanoma patients, respectively (Fig. S3). Nitric oxide synthase (NOS) was shown to be increased in metastatic melanoma cells, which is in accordance with our results [31].

The expression patterns of hypertension-related genes varied across different cancer types. The expression patten of hypertension-related gene also has prognostic potential in clinical practice (Fig. 3). To further explore the clinical implications of the hypertension-related genes, the relationships between these genes and CAGs were calculated. The results showed that the expression profiles of hypertension-related genes were correlated with CAGs, which may be associated with protein–protein interaction (Fig. 4A). A protein–protein interaction between *HRAS* (which encodes RASH protein) and *AGTR1* (which encodes AGTR1 protein) was observed.

We next focused on the biological pathways associated with hypertension-related genes. Hypertension-related genes were positively correlated with the EMT, Hormone AR, and RTK related pathways, whereas they were negatively correlated with the apoptosis, cell cycle, and DNA damage response related signaling pathways (Fig. 5). These pathways have been shown to play important roles in different types of cancer [32–35].

Finally, we calculated the correlation between hypertension-related genes and drug efficiency. The hypertension-related genes were related to the efficiency of multiple anti-cancer drugs (Fig. 6). *NOS3* was positively correlated and *INSR* was negatively correlated with the resistance of cancer to the majority of anti-cancer drugs. Some other genes were correlated with the resistance or sensitivity of cancers to certain drugs. These drug efficiency–correlated genes are of potential clinical significance since they may act as predictors of drug sensitivity. In accordance with our findings, previous studies have shown that *NOS3* and *AGTR1* are related to the chemoresistance of cancer [36–38]: *NOS3* is related to oxaliplatin resistance in colorectal cancer cells [36], and *AGTR1* is a marker of the drug resistance of breast cancer [37, 38].

## Conclusion

We systematically characterized and highlighted the critical role of hypertension-related genes in cancer. The hypertension-related genes are shown to play important roles in cancer progression and may be promising therapeutic targets for cancer treatment and side effect management. Our results may provide new insights into the clinical management of cancer combined with cardiovascular disorders.

## Supplementary Information

**Additional file 1:** **Fig. S1.** Significant homozygous amplification and deletion peaks of hypertension-related genes for each cancer type. The dot size represents the percentage of CNA. Red and blue dots represent the amplification peak and deletion peak, respectively. Dot size represents the homozygous percentage of CNA (Hete CAN %). Red and blue dots represent the amplification peak and deletion peak, respectively

**Additional file 2:** **Fig. S2** The correlation between the copy number alteration of hypertension-related genes and cancer. Dot size represents statistical significance. Dot colour represents Pearson’s correlation coefficient

**Additional file 3:** **Fig. S3** The correlation between single nucleotide variants of hypertension-related genes and overall survival of cancer patients. Dot size represents statistical significance. Blue and red represent negative and positive correlation with poor survival

**Additional file 4:** **Fig. S4** The single nucleotide variant (SNV) characteristics of hypertension-related genes in different types of cancer. **A** Variant type. SNP is short for single nucleotide polymorphism. Ins and Del are short for insertion and deletion, respectively. **B** Variant classification. **C** SNV class. **D** Variants per sample. **E** Variant classification summary. **F** Frequently mutated genes

## Data Availability

The data that support the findings of this study are available from the corresponding author upon request.

## Abbreviations

ADD1: α-Adducin
AR: Androgen receptor
CAG: Clinically actionable gene
CNA: Copy number alteration
EMT: Epithelial to mesenchymal transition
FDR: False discovery rate
GSEA: Gene set enrichment analysis
OS: Overall survival
PCC: Pearson’s correlation coefficient
PPI: Protein–protein interaction
RTK: Receptor tyrosine kinase
SNV: Single nucleotide variant
TCGA: The cancer genome atlas
