# Supplementary figures and images for: Comprehensive molecular characterization of hypertension-related genes in cancer
Source: Cardiooncology. 2022 May 5;8:10. doi: 10.1186/s40959-022-00136-z (PMC9069779; doi:10.1186/s40959-022-00136-z)

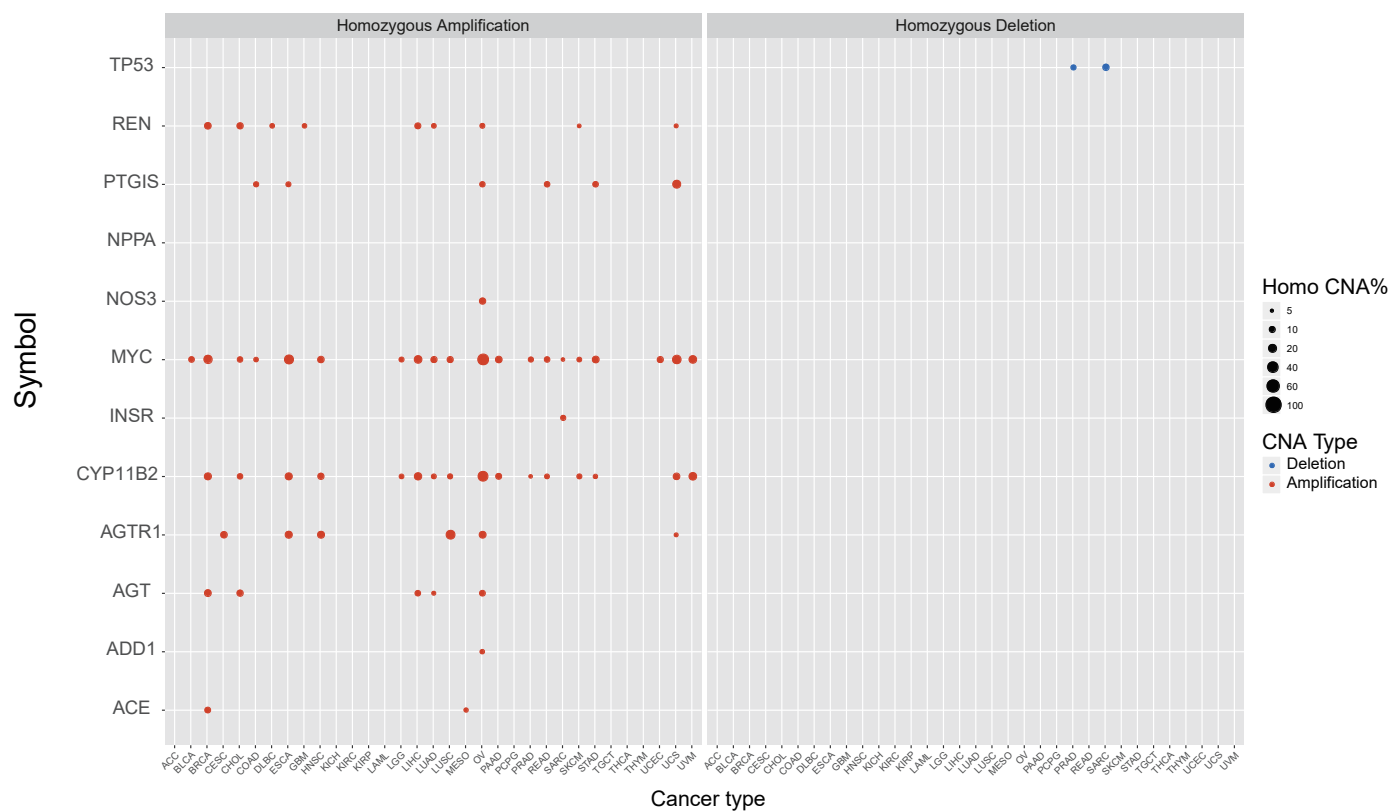

Supplement: Supplementary file 1 — Additional file 1: Fig. S1. Significant homozygous amplification and deletion peaks of hypertension-related genes for each cancer type. The dot size represents the percentage of CNA. Red and blue dots represent the amplification peak and deletion peak, respectively. Dot size represents the homozygous percentage of CNA (Hete CAN %). Red and blue dots represent the amplification peak and deletion peak, respectively [file 40959_2022_136_MOESM1_ESM.pdf]

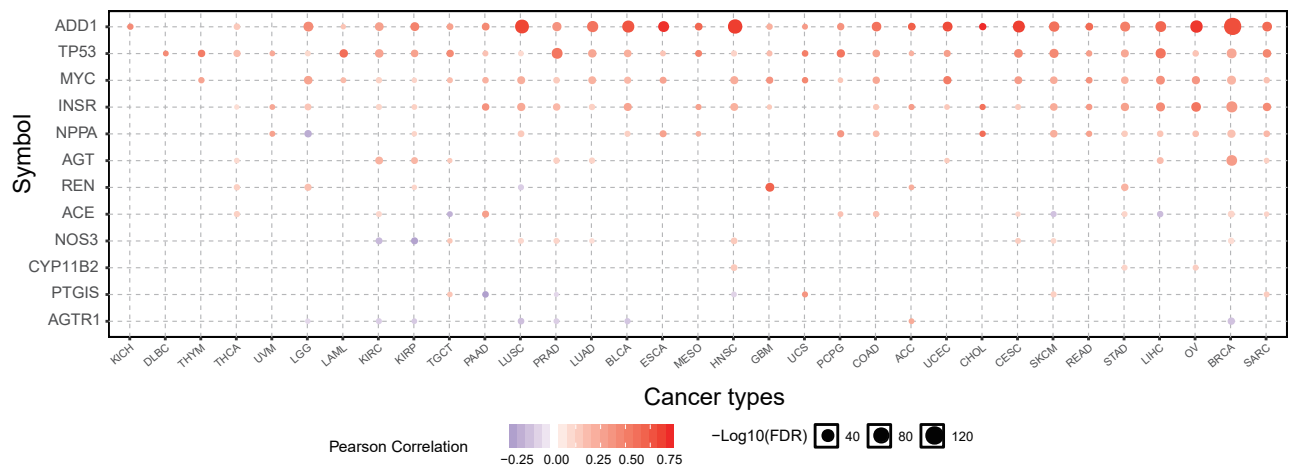

Supplement: Supplementary file 2 — Additional file 2: Fig. S2 The correlation between the copy number alteration of hypertension-related genes and cancer. Dot size represents statistical significance. Dot colour represents Pearson’s correlation coefficient [file 40959_2022_136_MOESM2_ESM.pdf]

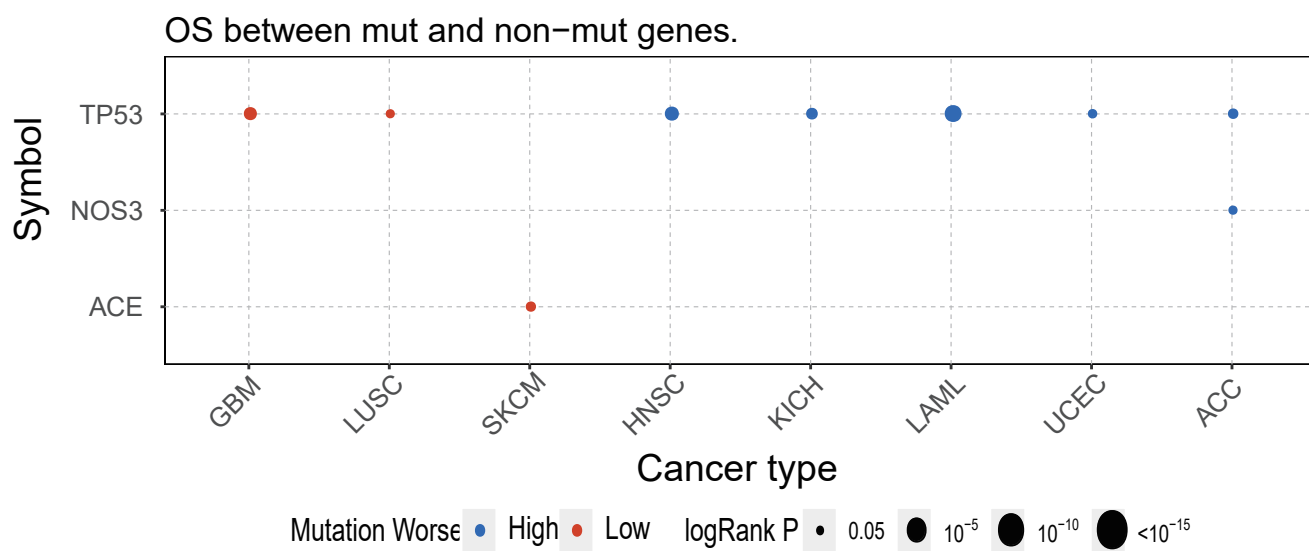

Supplement: Supplementary file 3 — Additional file 3: Fig. S3 The correlation between single nucleotide variants of hypertension-related genes and overall survival of cancer patients. Dot size represents statistical significance. Blue and red represent negative and positive correlation with poor survival [file 40959_2022_136_MOESM3_ESM.pdf]

A

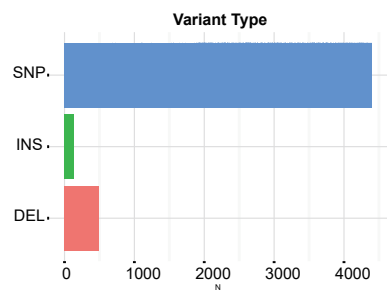

B

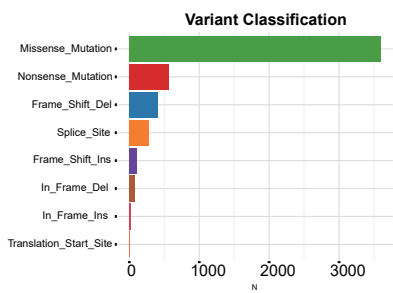

C

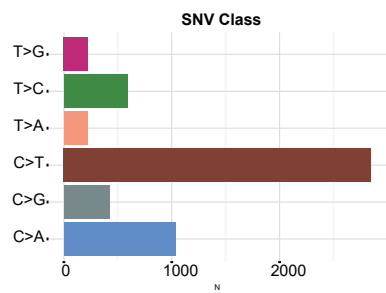

D

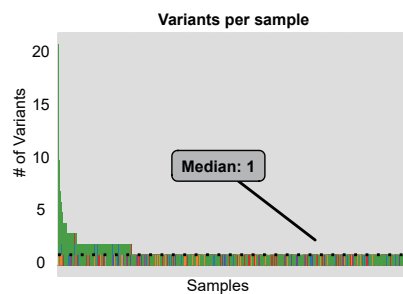

E

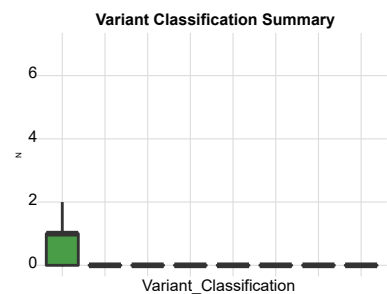

F

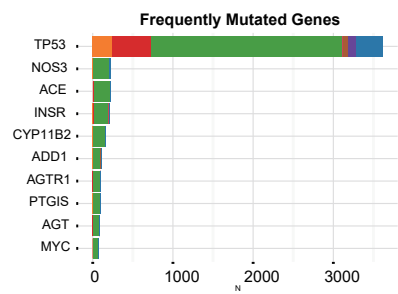

Supplement: Supplementary file 4 — Additional file 4: Fig. S4 The single nucleotide variant (SNV) characteristics of hypertension-related genes in different types of cancer. A Variant type. SNP is short for single nucleotide polymorphism. Ins and Del are short for insertion and deletion, respectively. B Variant classification. C SNV class. D Variants per sample. E Variant classification summary. F Frequently mutated genes [file 40959_2022_136_MOESM4_ESM.pdf]
